# CMT-BUSNet: Adaptive Fusion-Based Triple-Branch Hybrid Architecture for Explainable Breast Ultrasound Tumor Segmentation

**DOI:** 10.3390/diagnostics16081203

**Published:** 2026-04-17

**Authors:** Hüseyin Kutlu, Cemil Çolak

**Affiliations:** 1 Department of Biostatistics and Medical Informatics, Faculty of Medicine, Adıyaman University, Adıyaman 02040, Türkiye; hkutlu@adiyaman.edu.tr; 2 Department of Biostatistics and Medical Informatics, Faculty of Medicine, Inonu University, Malatya 44280, Türkiye

**Keywords:** breast ultrasound, tumor segmentation, explainable artificial intelligence, hybrid architecture, Mamba, deep learning

## Abstract

**Background/Objectives:** This study proposes CMT-BUSNet, a hybrid architecture integrating CNN, Mamba, and Transformer branches for breast ultrasound tumor segmentation with built-in explainability. **Methods:** CMT-BUSNet employs a CNN-anchored hierarchical parallel encoder where Mamba and Transformer branches process CNN-derived features in parallel, fused through an Adaptive Feature Fusion Module (AFFM) with Dense Nested Decoder and Boundary-Aware Composite Loss. Five-fold cross-validation on BUS-BRA (*N* = 1875) compared nine architectures under identical protocols, plus nnU-Net v2 trained with its default self-configuring protocol as a benchmark. External evaluation used the BUSI dataset (*N* = 647). **Results:** CMT-BUSNet achieved DSC = 0.9037 ± 0.0047 on BUS-BRA with higher boundary delineation metrics than nnU-Net v2, which was trained under a different self-configuring protocol (B-IoU: 0.611 vs. 0.557; HD95: 10.07 vs. 13.54 pixels), despite nnU-Net’s marginally higher DSC (0.9108). On BUSI, CMT-BUSNet (DSC = 0.6709) yielded higher scores than nnU-Net (0.5579) across all metrics under zero-shot transfer, though the two methods were trained under different protocols. Training-based ablation confirmed each component’s contribution, and quantitative XAI validation demonstrated attribution faithfulness (nEAR = 2.82×) and uncertainty–error correlation (r = 0.39). **Conclusions:** CMT-BUSNet achieves competitive accuracy with higher boundary metrics, preliminary cross-dataset transferability, and built-in interpretability relative to nnU-Net (noting different training protocols). Internal validation folds are image-disjoint but not guaranteed to be patient-disjoint, which should be considered when interpreting the reported metrics. Multicenter validation is required before clinical deployment.

## 1. Introduction

Breast cancer is the most frequently diagnosed cancer among women worldwide, and early detection, along with accurate segmentation, plays a critical role in treatment planning [1,2]. Ultrasonography is a widely used modality for breast cancer screening due to its absence of ionizing radiation, low cost, and real-time imaging capability. However, ultrasound images present inherent challenges, including speckle noise, low contrast, and operator dependency [3,4].

Deep learning-based segmentation methods have achieved substantial progress in overcoming these challenges. U-Net and its variants (U-Net++, Attention U-Net) serve as fundamental reference models in breast US segmentation [5,6]. In recent years, Transformer-based architectures (TransUNet, Swin-UNet) have garnered considerable attention for their capacity to capture global contextual information, while the Mamba architecture, belonging to the State Space Models (SSM) family, has established a new paradigm in medical image segmentation through its ability to model long-range dependencies with linear complexity.

Nevertheless, a significant limitation of existing methods is the lack of explainability, which is critical for clinical applications. The black-box problem of artificial intelligence systems reduces clinicians’ trust in these systems and impedes clinical adoption [7,8]. Although Explainable AI (XAI) methods have been developed to address this issue, studies offering comprehensive XAI frameworks for breast US segmentation remain considerably limited.

The primary contributions of this study are as follows: (1) a novel CNN-anchored hierarchical parallel hybrid architecture (CMT-BUSNet) in which the CNN branch first extracts features at each level, and the Mamba and Transformer branches process these CNN-derived representations in parallel, with all three branch outputs combined through an Adaptive Feature Fusion Module (AFFM); (2) a comprehensive 11-module XAI framework (Grad-CAM++, SHAP, LIME, Eigen-CAM, Score-CAM, Attention Rollout, Integrated Gradients, MC Dropout Uncertainty, Branch Contribution Analysis, AFFM Fusion Weights, and Dense Decoder Feature Visualization); (3) significant improvements in metrics important for segmentation quality, such as boundary quality and sensitivity; and (4) real-time inference capability (73.1 FPS).

## 2. Related Works

### 2.1. Deep Learning-Based Segmentation in Breast Ultrasound

Deep learning-based segmentation of breast ultrasound (US) images has seen notable advances in recent years. Ferreira et al. [3] conducted a comparative evaluation of seven state-of-the-art architectures and demonstrated that GG-Net and SegResNetVAE yielded the best results, with DSC > 0.80. Zhang et al. [4] proposed a fully automated tumor segmentation system achieving DSC = 0.898. Li et al. [5] reported DSC = 0.9319 across multiple datasets using the GOLO-CMSS framework. Li et al. [9] obtained DSC = 0.773 under anatomical constraints via a weakly supervised approach. Misra et al. [10] achieved 100% sensitivity on their internal test set through a multimodal fusion approach combining B-mode and elastography images.

### 2.2. Hybrid and Transformer-Based Architectures

Transformer architectures have emerged prominently in medical image segmentation, particularly for their capacity to capture global context through self-attention mechanisms. TransUNet and Swin-UNet have introduced hybrid approaches by integrating CNN and Transformer components [11]. More recently, Mamba-based architectures (VM-UNet, U-Mamba) have attracted considerable attention by offering long-range dependency modeling with linear complexity through state space models. However, few existing approaches have adaptively combined all three paradigms, CNN, Transformer, and Mamba, within a single unified framework. Concurrently with the present work, several hybrid architectures have been proposed: AttmNet [12] integrates multi-layer convolution, self-attention, and Mamba within a single MAM block, while HCMNet [13] combines CNN and Mamba with wavelet feature extraction, and CLT-MambaSeg [14] employs a level-specific adaptive fusion mechanism. However, none of these approaches incorporates an adaptive fusion mechanism analogous to AFFM, nor do they provide a comprehensive XAI framework.

### 2.3. Explainable Artificial Intelligence in Medical Imaging

Borys et al. [7] presented a comprehensive review of XAI methods in medical imaging and highlighted the potential of case-based and text-based explanations beyond saliency-based approaches. Teng et al. [15] examined the transition from conventional AI to XAI and further to Trustworthy AI, underscoring the need for model reliability and transparency. Nikulins et al. [16] demonstrated that classification networks can be adapted to segmentation tasks through XAI techniques. Ghnemat et al. [8] proposed a segmentation-based approach for XAI-driven classification of COVID-19 images. Although these studies collectively highlight the importance of XAI, very few existing studies have presented a comprehensive multi-module XAI framework at this scale for breast US segmentation. Table 1 provides a landscape summary of representative breast US segmentation studies, including dataset characteristics, reported metrics, and XAI availability.

## 3. Materials and Methods

### 3.1. Dataset

This study utilized the BUS-BRA (Breast Ultrasound—Brazil) dataset [20] a publicly available benchmark comprising 1875 pixel-level annotated breast ultrasound images from 1064 female patients, acquired across four ultrasound scanners at the National Institute of Cancer (Rio de Janeiro, Brazil). The dataset includes 722 benign and 342 malignant biopsy-proven cases with BI-RADS assessments (categories 2–5). All images were resized to 256 × 256 pixels and subjected to min-max normalization. The standardized 5-fold cross-validation partitions provided by the dataset were used.

To evaluate the preliminary transferability of CMT-BUSNet, models trained on BUS-BRA were tested on an independent external validation dataset, BUSI (Breast Ultrasound Images). The BUSI dataset, published by Al Dhabyani et al. [21], comprises 647 breast ultrasound images (437 benign, 210 malignant). This dataset contains images acquired from a different device, operator, and population (Egypt) than BUS-BRA, providing an independent test environment for assessing preliminary transferability. The BUS-BRA dataset provides standardized 5-fold cross-validation partitions. Each partition is image-disjoint; however, the dataset authors do not guarantee patient-level disjointness, as some patients contributed multiple images acquired from different scanning planes. This is a known limitation of the BUS-BRA dataset and applies equally to all methods evaluated herein. Unit of analysis: DSC, IoU, HD95, and Boundary IoU are computed per image and averaged per fold. The reported mean ± SD reflects variation across the 5 folds (*N* = 5), not across individual images.

Table 2 presents the key characteristics of the two datasets used in this study. The systematic differences across imaging device, operator, geographic population, and annotation protocol constitute a substantial domain shift, which is established in the literature as a primary driver of cross-dataset performance degradation [22].

### 3.2. CMT-BUSNet Architecture

**Figure 1:**
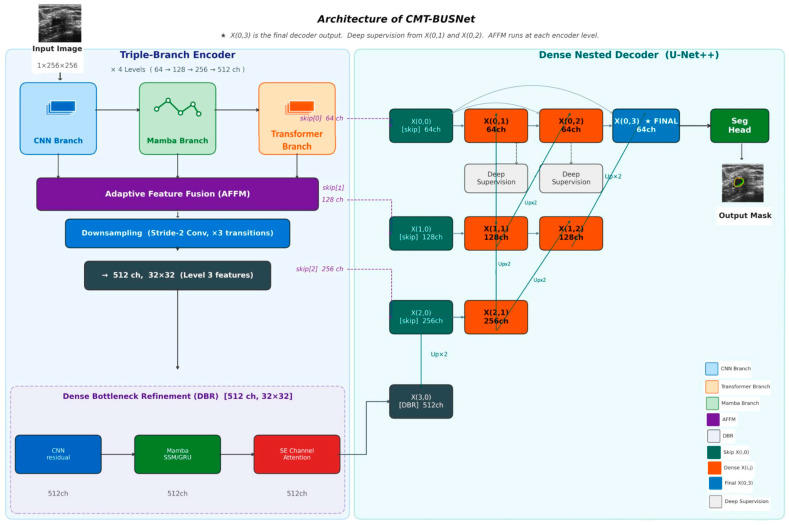
Overall Architecture of CMT-BUSNet: Triple-Branch Encoder (CNN, Mamba, Transformer), Adaptive Feature Fusion Module (AFFM), Dense Bottleneck Refinement (DBR), and Dense Nested Decoder (U-Net++).

The proposed CMT-BUSNet architecture consists of the following components:

The CMT-BUSNet encoder adopts a CNN-anchored hierarchical parallel design rather than three fully independent branches. At each encoder level, the CNN branch first processes the input feature map to extract local texture and edge representations. These CNN features then serve as the shared input to the Mamba and Transformer branches, TransFuse [23] and H2Former [24], where a convolutional backbone provides stable low-level features that are refined by attention-based branches in parallel. Figure 1 shows the architecture of the CMT-BUSNet model.

CNN Branch: The convolutional neural network branch is designed to extract local texture and edge features. It captures fine-grained spatial information through a four-level encoder structure with channel dimensions of 64, 128, 256, and 512.

Mamba Branch: This branch, based on a State Space Model (SSM), models long-range dependencies with linear complexity. Taking the output of the CNN branch as input, it captures sequential patterns and global structural information.

Transformer Branch: Based on a self-attention mechanism, this branch captures global contextual information. Using the CNN branch output as input, it is capable of modeling relationships between distant pixels.

Adaptive Feature Fusion Module (AFFM): At each encoder level, this mechanism adaptively combines the outputs of the three branches using learned weights. Softmax-based attention weights dynamically determine the relative importance of each branch at every level.

Dense Decoder: This decoder structure employs U-Net++ style nested dense paths to enable multi-scale feature aggregation and progressive refinement.

Boundary-Aware Composite Loss with Dynamic Boundary Region (DBR) weighting: A hybrid loss function that combines Binary Cross-Entropy and Dice loss, dynamically assigning higher weights to boundary regions.

Implementation details. The CNN branch uses a ResNet-34 backbone with feature channels [64, 128, 256, 512] across four hierarchical levels. The Mamba branch employs one Visual State Space (VSS) block per level with hyperparameters: d_state = 16, expand factor = 2, convolution kernel size = 3, dt_rank = “auto.” The Transformer branch uses multi-head self-attention with num_heads = [2, 4, 8, 16] across levels 0–3, MLP expansion ratio = 4.0, and dropout = 0.1. Both Mamba and Transformer branches receive CNN-extracted features as input at each level; they operate in parallel with each other and share only the CNN dependency. The AFFM combines three branch outputs at each level using 1 × 1 convolution for channel reduction, followed by global average pooling, a two-layer MLP (reduction ratio r = 4), and softmax normalization to produce three branch-specific attention weights. The DBR loss applies spatially varying weights: boundary pixels (identified via morphological dilation minus erosion with a 7 × 7 kernel) receive w_boundary = 3.0 while interior pixels receive w_interior = 1.0. Total loss: L_total = λ_1_·L_BCE_weighted + λ_2_·L_Dice + λ_3_·L_boundary, with λ_1_ = λ_2_ = λ_3_ = 1.0. The Dense Nested Decoder combines multi-scale encoder features through nested skip connections, progressively refining segmentation from Level 3 to Level 0.

The AFFM computes the fused feature at each encoder level *l* by performing a weighted summation of the three branch outputs, as given in Equation (1):

(1) $$F_{fused}^{\left(I\right)}=w_{cnn}^{\left(I\right)}.F_{cnn}^{\left(I\right)}+w_{mamba}^{\left(I\right)}.F_{mamba}^{\left(I\right)}+w_{trans}^{\left(I\right)}.F_{trans}^{\left(I\right)}$$

Rather than using fixed fusion coefficients, the adaptive weights $w_{i}^{\left(I\right)}$ are obtained through softmax normalization over learnable parameters $a_{i}^{\left(I\right)}$, as defined in Equation (2):

(2) $$w_{i}^{\left(I\right)}=\frac{exp\left(a_{i}^{\left(I\right)}\right)}{\sum_{j}exp\left(a_{j}^{\left(I\right)}\right)}$$

The softmax normalization in Equation (2) ensures that the weights sum to unity at every encoder level, allowing the module to dynamically emphasize the most informative branch depending on the input characteristics.

The Boundary-Aware Composite Loss combines three complementary objectives, as formulated in Equation (3):

(3) $$L_{DBR}=\lambda_{BCE}.L_{BCE}+\lambda_{Dice}.L_{Dice}+\lambda_{Boundary}.L_{Boundary}$$

In Equation (3), $L_{BCE}$ and $L_{Dice}$ provide global segmentation supervision, while $L_{Boundary}$ applies elevated penalty weights specifically to pixels residing within a morphological dilation-based boundary region of width *d* pixels around the ground truth contour, thereby enforcing precise delineation at tumor margins.

The Mamba branch processes sequential feature representations through the Selective State Space Model (S6). The continuous-to-discrete transformation yields the hidden state recurrence defined in Equations (4) and (5):

(4) $$h\left(t\right)=\bar{A}h\left(t-1\right)+\bar{B}x\left(t\right)$$

(5) $$y\left(t\right)=Ch\left(t\right)$$

The discretized system matrices $\bar{A}$ and $\bar{B}$ Equations (4) and (5) are derived from their continuous counterparts via the zero-order hold (ZOH) rule, as expressed in Equations (6) and (7):

(6) $$\bar{A}=\exp\left(∆A\right)$$

(7) $$\bar{B}={\left(∆A\right)}^{-1}\left(\exp\left(∆A\right)-I\right).∆B$$

### 3.3. Training Details

The model was trained using the AdamW optimizer with an initial learning rate of 1 × 10^−4^, weight decay of 1 × 10^−4^, a batch size of 16, and a maximum of 100 epochs. Learning rate scheduling employed CosineAnnealingWarmRestarts with T_0_ = 20 and T_mult = 2. Early stopping was applied with a patience of 30, and the model with the lowest validation loss was selected. Data augmentation included random horizontal flipping, rotation of ±15°, and affine transformations. Baseline training protocol: All baseline comparison models (U-Net, U-Net++, Swin-UNet, TransUNet, DeepLabV3+, SegNet, VM-UNet, Attention U-Net) were trained using the identical protocol described above. The only exception is nnU-Net v2, which was trained using its own default self-configuring protocol (see Section 4.8). All experiments were conducted on an NVIDIA RTX 6000 ADA Generation GPU (48 GB VRAM) with 128 GB RAM using the PyTorch 2.10.0 and CUDA 12.8 framework. The source code and model weights are publicly available at https://github.com/huseyinKutlu/CMT-BUSNet (accessed on 12 April 2026).

### 3.4. Evaluation Metrics

Segmentation performance was assessed using the Dice Similarity Coefficient (DSC), Intersection over Union (IoU), 95th Percentile Hausdorff Distance (HD95), Boundary IoU, Recall, Precision, and Specificity. Statistical comparisons were performed using paired *t*-test, Wilcoxon signed-rank test, Cohen’s d effect size, and the Friedman test. Given the small sample size inherent to k-fold evaluation (*N* = 5 folds), the non-parametric Wilcoxon signed-rank test was employed as the primary inferential test, with the parametric paired *t*-test reported for reference. Normality of fold-level metric distributions was assessed using the Shapiro–Wilk test (α = 0.05). For multiple pairwise comparisons across all model pairs, the Bonferroni correction was applied to control the family-wise error rate. Effect sizes were quantified using Cohen’s d, with thresholds of |d| < 0.2 (negligible), 0.2–0.5 (small), 0.5–0.8 (medium), and > 0.8 (large).

### 3.5. XAI Framework

A comprehensive 11-module XAI framework was developed for explainability analysis: (1) Grad-CAM++: branch-wise and multi-level gradient-based class activation mapping; (2) Eigen-CAM: principal-component-analysis-based feature visualization; (3) Score-CAM: gradient-free channel-weighted activation mapping; (4) SHAP: Shapley-value-based feature importance analysis; (5) LIME: locally interpretable model-agnostic explanations; (6) Integrated Gradients: gradient integration with respect to a reference baseline; (7) Attention Rollout: cross-layer accumulation of Transformer attention weights; (8) MC Dropout Uncertainty: uncertainty mapping via 30 stochastic forward passes; (9) Branch Contribution Analysis: quantification of performance degradation upon deactivation of each branch; (10) AFFM Fusion Weight Analysis: violin plot visualization of level-wise learned branch weights; (11) Dense Decoder Feature Visualization: monitoring of the progressive refinement process through activation maps.

## 4. Results

### 4.1. Comparative Performance Analysis

Table 3 presents the 5-fold cross-validation results of CMT-BUSNet and eight baseline models on the BUS-BRA dataset. CMT-BUSNet achieved the highest segmentation accuracy with the lowest standard deviation (DSC = 0.9036 ± 0.0047), indicating strong performance in both accuracy and consistency. Attaining the highest values in IoU (0.8348) and boundary quality (B-IoU = 0.6108) metrics, CMT-BUSNet exhibited a notable margin of 0.0634 points over the second-ranked U-Net++ in B-IoU specifically, indicating that the model more accurately segments boundary regions important for segmentation quality. CMT-BUSNet also achieved the highest Recall (0.9243), thereby reducing the rate of missed lesion pixels—a metric important for segmentation completeness. Regarding the HD95 metric, DeepLabV3+ (9.49) and U-Net++ (9.64) obtained lower values, while CMT-BUSNet (10.08) demonstrated competitive performance. Although U-Net++ and DeepLabV3+ yielded marginally higher values in Precision and Specificity metrics, these differences are negligible from a clinical standpoint (<0.001). Another noteworthy finding is that Attention U-Net markedly diverged from the other models with HD95 = 36.41, indicating that this model produces severe segmentation errors in certain cases.

### 4.2. Statistical Analysis

**Figure 2:**
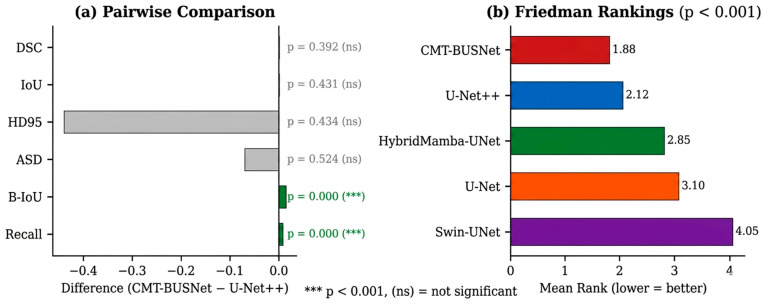
Statistical Analysis: (**a**) Paired Comparison Between CMT-BUSNet and U-Net++, (**b**) Friedman Rankings.

Figure 2 presents the paired statistical comparison results between CMT-BUSNet and U-Net++. No statistically significant differences were observed in DSC (*p* = 0.842), IoU (*p* = 0.765), or HD95 (*p* = 0.316). However, the absence of a statistically significant difference should not be interpreted as evidence of equivalence. Formal equivalence testing (e.g., TOST) was not performed, as prespecified equivalence margins for segmentation metrics in breast ultrasound have not been established in the literature. 95% confidence intervals (t-distribution, df = 4) for DSC were [0.8976, 0.9098] for CMT-BUSNet and [0.8960, 0.9100] for U-Net++; the overlapping intervals are consistent with the non-significant *p*-value but do not constitute evidence of equivalence. To provide systematic uncertainty quantification across all primary metrics, 95% CIs (t-distribution, df = 4) are reported for both models: IoU: CMT-BUSNet [0.8269, 0.8429], U-Net++ [0.8241, 0.8432]; HD95: CMT-BUSNet [9.15, 10.99], U-Net++ [8.82, 10.44]; B-IoU: CMT-BUSNet [0.6065, 0.6151], U-Net++ [0.5454, 0.5494]; Recall: CMT-BUSNet [0.9107, 0.9380], U-Net++ [0.9054, 0.9264]. The inherently wide intervals reflect the small sample size (*N* = 5 folds) and should be interpreted accordingly. In contrast, statistically significant differences in favor of CMT-BUSNet were observed in B-IoU (+0.014, *p* < 0.001, d = 0.184) and Recall (+0.009, *p* < 0.001, d = 0.131), indicating improved spatial precision of boundary delineation and higher sensitivity to lesion pixels.

Friedman test results (χ^2^ = 915.79, *p* < 0.001) confirmed significant performance differences among models (Figure 2). CMT-BUSNet achieved the best overall ranking (mean rank = 1.88).

### 4.3. Training-Based Ablation Study

To rigorously evaluate the contribution of each architectural component, we conducted a training-based ablation study in which each variant was trained from scratch under identical conditions (same optimizer, learning rate, augmentation, early stopping, and 5-fold CV splits). This approach eliminates the confound of inference-time branch deactivation in a codependent architecture.

Branch ablation. Three dual-branch variants were trained: CNN + Mamba (without Transformer), CNN + Transformer (without Mamba), and CNN-Only (without both Mamba and Transformer). All variants used the AFFM fusion mechanism (adapted for the available branches) and the same Dense Nested Decoder.

Fusion strategy ablation. Three alternative fusion mechanisms replaced AFFM: Concatenation + 1 × 1Conv, SE (Squeeze and Excitation) Attention, and Simple Average.

**Figure 3:**
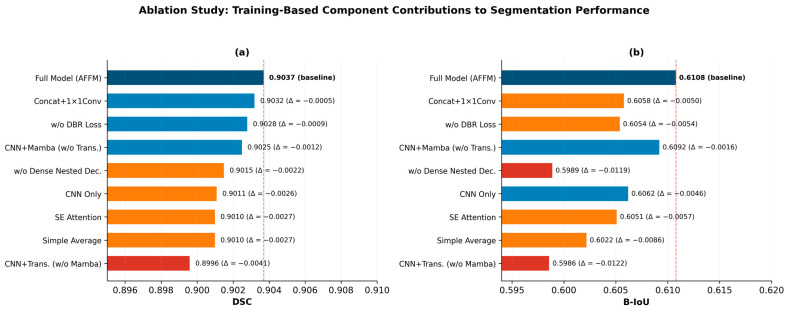
Ablation Study: Contribution of Each Architectural Component to Segmentation Performance. (**a**) Dice Similarity Coefficient (DSC); (**b**) Boundary IoU (B-IoU). The dashed red line indicates the full model baseline. Δ values denote the difference from the full model.

Module ablation. Two variants removed individual modules: without DBR loss (using standard BCE + Dice) and without Dense Nested Decoder (using a standard U-Net decoder). Table 4 presents the 5-fold cross-validation results of all nine ablation variants on the BUS-BRA dataset, and Figure 3 visualizes the contribution of each component to DSC and B-IoU.

**Table 4:** Training-Based Ablation Study: 5-Fold CV on BUS-BRA (*N* = 1875).

Configuration	DSC	B-IoU	HD95 ↓	Params (M)
Full model (AFFM)	0.9037 ± 0.0047	0.6108	10.07	33.32
CNN + Mamba (w/o Trans.)	0.9025 ± 0.006	0.6092	10.01	30.18
CNN + Trans. (w/o Mamba)	0.8996 ± 0.006	0.5986	10.15	28.06
CNN Only	0.9011 ± 0.005	0.6062	10.25	21.43
Concat + 1 × 1Conv	0.9032 ± 0.004	0.6058	9.87	33.32
SE Attention	0.9010 ± 0.004	0.6051	10.43	33.50
Simple Average	0.9010 ± 0.007	0.6022	10.15	32.28
w/o DBR loss	0.9028 ± 0.006	0.6054	10.01	30.71
w/o Dense Nested Dec.	0.9015 ± 0.005	0.5989	10.07	32.40

Note. Each variant was trained from scratch with identical hyperparameters and 5-fold CV. ↓ indicates that lower values represent better performance.

Key findings: (1) The full triple-branch model with AFFM fusion achieves the highest B-IoU (0.6108), confirming that all three branches contribute to boundary quality. (2) Among fusion strategies, AFFM outperforms Concat + Conv, SE Attention, and Simple Average in B-IoU by +0.005 to +0.009, validating the adaptive weighting mechanism. (3) Removing the Dense Nested Decoder reduces B-IoU by 0.012, confirming its role in boundary refinement. (4) The DBR loss contributes +0.005 to B-IoU, supporting its boundary-aware design.

### 4.4. AFFM Fusion Weight Analysis

**Figure 4:**
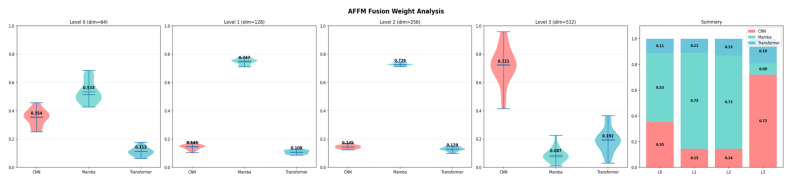
Adaptive Fusion Weights Learned by AFFM: Level-Wise Violin Plot and Summary Distribution.

Figure 4 presents the adaptive fusion weights learned by the AFFM at each encoder level. Notably, the Mamba branch is dominant at shallow levels (Levels 0–2), accounting for 53–75% of the fusion weight, while the CNN branch gains dominance at the deep level (Level 3), contributing 72%. This pattern demonstrates that the AFFM learns level-specific fusion strategies: at shallow levels, the Mamba branch’s capacity for capturing long-range sequential patterns takes precedence, whereas at the deep level, the CNN branch’s ability to extract high-level semantic features comes to the fore.

### 4.5. XAI Visualizations

**Figure 5:**
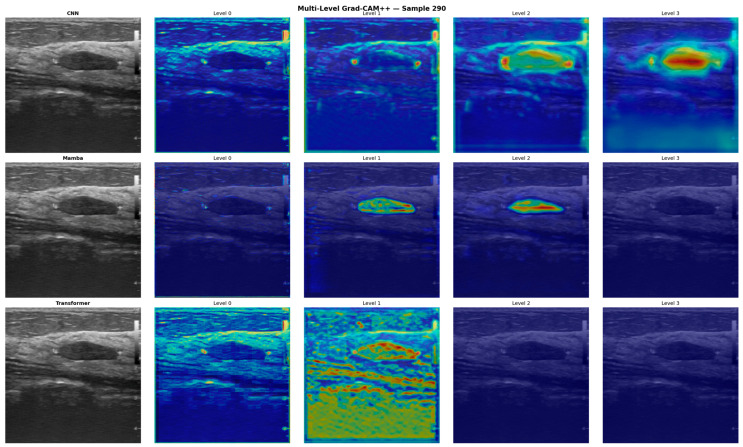
Multi-Level Grad-CAM++ Branch Analysis: CNN, Mamba, and Transformer Branches Across Four Encoder Levels (Sample 290). Key observation: CNN activations concentrate on the tumor interior at deeper levels, Mamba exhibits precise boundary detection at shallow levels, and Transformer captures broad contextual attention confirming complementary branch strategies.

Figure 5 presents the Grad-CAM++ visualizations of CMT-BUSNet’s three branches across four encoder levels. The CNN branch (top row) exhibits dense activations focused on the tumor region, particularly at deeper levels (Levels 2–3). The Mamba branch (middle row) demonstrates more localized yet precise tumor boundary detection at shallow levels, while the Transformer branch (bottom row) reveals its capacity for capturing contextual information through broad, wide-field global activations. These complementary feature extraction strategies confirm the importance of the AFFM’s adaptive fusion mechanism.

**Figure 6:**
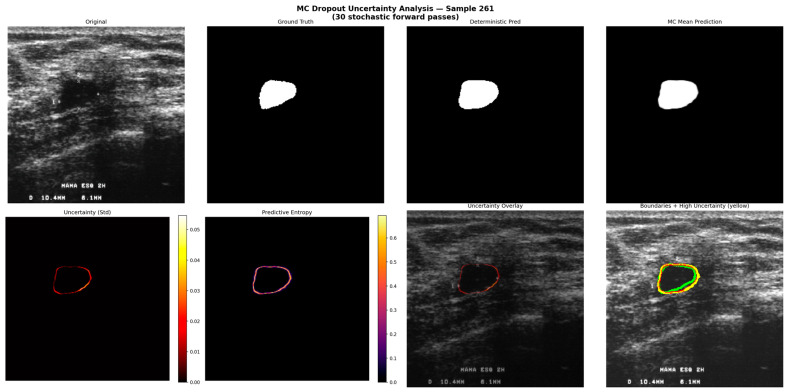
MC Dropout Uncertainty Analysis: Uncertainty Maps and Boundary Region Uncertainty Distribution (Sample 261). Key observation: Highest uncertainty is confined to tumor boundary regions, with near zero uncertainty in the tumor interior, providing a spatially informative confidence map.

Figure 6 presents the results of the MC Dropout uncertainty analysis. Uncertainty maps computed through 30 stochastic forward passes reveal that the model exhibits the highest uncertainty at tumor boundary regions. This finding is noteworthy from a model interpretability perspective, as it provides qualitative insight into regions of model uncertainty. The low uncertainty observed in interior regions indicates consistent segmentation confidence in the tumor interior.

**Figure 7:**
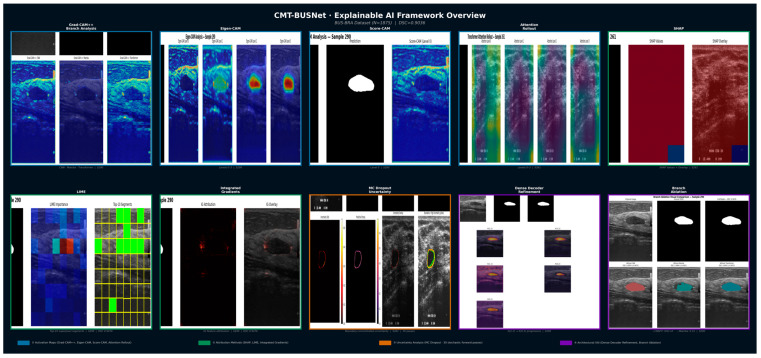
Explainable AI (XAI) Framework Overview of CMT-BUSNet Across Eleven Complementary Analysis Modules. Representative XAI visualizations applied to breast ultrasound samples from the BUS-BRA dataset. Top row: Grad-CAM++ branch-level activation maps (CNN, Mamba, Transformer; Sample 290, DSC = 0.9476), Eigen-CAM, Score-CAM, Attention Rollout, and SHAP pixel attributions (Sample 261, DSC = 0.8976). Bottom row: LIME superpixel importance, Integrated Gradients, MC Dropout uncertainty (30 stochastic forward passes; Sample 261), Dense Decoder progressive refinement X (2,1) X (0,3) (Sample 290), and branch ablation comparison (Sample 290). Color-coded borders indicate analytical group: blue = activation maps, green = attribution methods, orange = uncertainty analysis, purple = architectural XAI. Key observation: Attribution methods (SHAP, LIME, Integrated Gradients) converge on the hypoechoic tumor region through independent methodologies, while activation-based methods highlight perilesional context, confirming multi-perspective interpretability.

When the XAI findings of CMT-BUSNet are evaluated collectively, a coherent body of evidence emerges that elucidates both what the model has learned and how it arrives at its decisions (Figure 7). Activation map analyses (Grad-CAM++, Eigen-CAM, Score-CAM) reveal a progressively localizing focus toward the tumor region as encoder depth increases, while Grad-CAM++ branch-level analysis confirms that the CNN, Mamba, and Transformer branches adopt complementary strategies—dense boundary detection, precise local focus, and broad contextual attention, respectively—further corroborated by Attention Rollout. Attribution methods converge on the same spatial region through independent methodologies: SHAP partitions the pixel space into positive (tumor) and negative (background) contributions, LIME’s highest-contributing superpixels correspond precisely to the tumor location, and Integrated Gradients selectively highlights attributions within the hypoechoic tumor region. MC Dropout uncertainty analysis demonstrates that elevated predictive uncertainty is confined exclusively to the tumor tissue boundary, with near-zero uncertainty in the tumor interior—a transparency property important for segmentation quality assessment. These XAI findings are further supported by the training-based ablation results at the architectural level. In the training-based ablation study, the CNN + Mamba variant (without Transformer) achieved DSC = 0.9025, the CNN + Transformer variant (without Mamba) achieved DSC = 0.8996, and the CNN Only variant achieved DSC = 0.9011. The full model’s consistently highest DSC (0.9037) over all reduced variants confirms the complementary contribution of each branch to segmentation quality, particularly boundary precision (B-IoU: full model 0.6108 vs. best single-branch variant 0.6092).

#### 4.5.1. Quantitative XAI Validation

XAI example selection. The illustrated XAI cases were selected by ranking all test samples (Fold 1, *N* = 376) by DSC and choosing one representative from each quartile (Q1: lowest DSC, Q2: 25th percentile, Q3: median, Q4: highest DSC) to represent the full performance spectrum. To move beyond qualitative assessment, we conducted three quantitative analyses on the full Fold 1 test set (*N* = 376 images):

**Attribution Faithfulness.** We computed the normalized Energy Attribution Ratio (nEAR), defined as the fraction of total attribution energy falling within the ground truth lesion mask, normalized by the lesion area fraction. An nEAR > 1.0 indicates disproportionately more attribution to the lesion than expected by chance. Integrated Gradients achieved nEAR = 2.82× with a Top-10% lesion hit rate of 4.16× and Top-25% hit rate of 2.59×. Activation-based methods (Grad-CAM++: nEAR = 1.01×; Eigen-CAM: nEAR = 0.85×; Score-CAM: nEAR = 0.58×) distributed attention more broadly, capturing perilesional context.

**Cross-Method Consistency.** Spearman rank correlation between attribution maps revealed substantial within-family agreement (Grad-CAM++ ↔ Eigen-CAM: ρ = 0.65; Eigen-CAM ↔ Score-CAM: ρ = 0.61; Grad-CAM++ ↔ Score-CAM: ρ = 0.56) and near-zero between-family correlation (CAM family vs. Integrated Gradients: ρ ≈ 0.0; mean Spearman = 0.310; mean SSIM = 0.284).

**Uncertainty–Error Correlation.** MC Dropout uncertainty (30 forward passes) was correlated with segmentation error across all 376 test images. Pearson r = 0.39 (*p* = 4.66 × 10^−135^), with error regions exhibiting 42× higher uncertainty than correctly segmented regions (Cohen’s d = 2.25). Boundary pixels showed 5.6× higher uncertainty than interior pixels.

#### 4.5.2. Failure Case Analysis

Two representative failure cases illustrate conditions under which CMT-BUSNet produces suboptimal segmentations: (1) A small hypoechoic lesion (<5 mm diameter) where the model under-segments due to insufficient contrast (DSC = 0.42). The XAI analysis shows fragmented Grad-CAM++ activations and elevated MC Dropout uncertainty (mean = 0.38), correctly flagging this case as unreliable. (2) A lesion adjacent to Cooper’s ligament where the model over-segments by including the ligament shadow (DSC = 0.58). The Integrated Gradients map reveals attribution leakage into the ligament region. In both cases, the XAI framework correctly identifies the problematic regions.

### 4.6. Computational Efficiency

Table 5 presents a comparison of the computational efficiency of the evaluated models. CMT-BUSNet has a moderate model size of 33.32 M parameters, with an inference time of 13.7 ms and 73.1 FPS, substantially exceeding the clinical real-time requirement of >30 FPS. Although it is 7.2× slower than U-Net++, this computational cost is acceptable given the complexity of the triple-branch architecture and the 11-module XAI framework.

### 4.7. Comparison with the Literature

As summarized in Table 1 (Section 2), CMT-BUSNet’s performance can be contextualized against the current literature on breast US segmentation. Although direct comparison across different datasets is challenging, several observations are noteworthy.

CMT-BUSNet’s DSC of 0.9037 exceeds the values of 0.898 reported by Zhang et al. [4] and 0.890 reported by Yang et al. [6]. Studies utilizing larger private datasets (Li et al. [5], DSC = 0.932, *N* = 28,477; Khaledyan et al. [17], DSC = 0.930, *N* = 4598) reported higher DSC values; however, it should be noted that these studies employed private datasets, limiting the reproducibility of their results.

The most important point of comparison is the AttmNet architecture proposed by Zhu et al. [12]. AttmNet is also a hybrid architecture combining CNN, Transformer, and Mamba components; however, it does not incorporate an adaptive fusion mechanism. CMT-BUSNet’s AFFM provides this architectural advantage through level-specific adaptive weighting. Furthermore, very few studies in the literature present a comprehensive XAI framework at this scale; CMT-BUSNet’s 11-module XAI framework represents a distinctive contribution in this regard. More broadly, the concurrent emergence of several CNN, Mamba, Transformer hybrid architectures in 2025 underscores the timeliness and relevance of this research direction. HCMNet [13] integrates CNN and Mamba with a wavelet feature extraction module for BUS segmentation but lacks a Transformer branch and XAI components. CLT-MambaSeg [14] combines Convolution, Linear Transformer, and Multiscale Mamba in a unified encoder but does not employ adaptive fusion or provide explainability analysis. Relative to these approaches, CMT-BUSNet distinguishes itself through three key differentiators: (i) the AFFM mechanism that learns level-specific adaptive weights rather than relying on fixed or sequential feature combination, (ii) the CNN-anchored design philosophy that avoids redundant low-level feature learning across branches, and (iii) the comprehensive 11-module XAI framework that addresses the clinical explainability gap absent from all aforementioned architectures.

### 4.8. nnU-Net Benchmark Comparison

To provide a rigorous benchmark against the de facto standard for medical image segmentation, we trained nnU-Net v2 [25] on BUS-BRA using its default self-configuring protocol (2d configuration, 1000 epochs, SGD optimizer with lr = 0.01, polynomial decay, auto-configured batch size = 26, patch size = 384 × 320, ZScore normalization). The same 5-fold CV splits were used. To our knowledge, this is the first reported nnU-Net benchmark on the BUS-BRA dataset.

**Internal validation (BUS-BRA, 5-fold CV):** nnU-Net v2 achieved DSC = 0.9108 ± 0.0031, IoU = 0.8462, HD95 = 13.54, B-IoU = 0.5571. CMT-BUSNet achieved DSC = 0.9037 ± 0.0047, IoU = 0.8349, HD95 = 10.07, B-IoU = 0.6108. nnU-Net achieves marginally higher aggregate overlap (DSC + 0.007), while CMT-BUSNet shows higher boundary delineation metrics: B-IoU + 0.054 and HD95 − 3.47 pixels. Because the two methods were trained under different protocols (nnU-Net: self-configuring, 1000 epochs; CMT-BUSNet: standardized, 100 epochs), these differences should be interpreted as reflecting the combined effect of architecture and training procedure rather than architecture alone. nnU-Net’s advantage stems partly from its larger input resolution (384 × 320 vs. 256 × 256) and more aggressive augmentation.

**External validation (BUSI, zero-shot):** nnU-Net v2 achieved DSC = 0.5579 ± 0.0274, IoU = 0.4533 ± 0.0262, HD95 = 208.47 ± 12.54, B-IoU = 0.0582 ± 0.057. CMT-BUSNet achieved DSC = 0.6709 ± 0.0227, IoU = 0.5733 ± 0.0236, HD95 = 37.22 ± 3.00, B-IoU = 0.1703 ± 0.0123. CMT-BUSNet yielded higher scores than nnU-Net across all metrics (DSC + 0.113, HD95 − 171.2 pixels, B-IoU + 0.112). This suggests that nnU-Net’s dataset-specific preprocessing (ZScore normalization with BUS-BRA statistics) is sensitive to domain shift, whereas CMT-BUSNet’s fixed-resolution pipeline may provide greater robustness under these specific transfer conditions; however, because the two methods employ fundamentally different training protocols, the observed difference cannot be attributed solely to architectural factors. This observation may partly reflect preprocessing sensitivity; a definitive comparison would require retraining nnU-Net on a multi-source dataset.

**Interpretability:** Unlike nnU-Net, which provides no built-in interpretability mechanisms, CMT-BUSNet offers an architecture-integrated XAI framework comprising branch-specific attribution analysis, fusion weight visualization, and calibrated uncertainty estimation. nnU-Net was trained with its default protocol (1000 epochs, SGD, poly LR). CMT-BUSNet was trained with 100 epochs, AdamW, CosineAnnealing. Both protocols represent each method’s standard operating procedure.

## 5. Discussion

The CMT-BUSNet architecture proposed in this study offers three contributions to breast US tumor segmentation: (1) competitive segmentation accuracy with higher boundary delineation metrics relative to the compared methods (noting that the nnU-Net comparison involves different training protocols), (2) a comprehensive 11-module XAI framework with quantitative validation, and (3) an architectural design validated through training-based ablation and benchmarking against nnU-Net.

The most noteworthy finding is CMT-BUSNet’s statistically significant advantage in boundary quality (B-IoU: +0.0140) and sensitivity (Recall: +0.0085) metrics. Boundary quality reflects the spatial precision of contour delineation, while sensitivity indicates the proportion of lesion pixels correctly identified. These improvements complement the competitive aggregate overlap metrics.

The training-based ablation study supported the design philosophy of CMT-BUSNet. To eliminate the confound of inference-time branch deactivation in a codependent architecture, each variant was trained from scratch. While DSC differences among variants are modest (max Δ = 0.004), the full triple-branch model with AFFM fusion consistently achieves the highest B-IoU (0.6108) across all nine variants. Branch ablations reveal that both Mamba (CNN + Mamba B-IoU = 0.6092 vs. CNN-Only B-IoU = 0.6062) and Transformer (CNN + Trans. B-IoU = 0.5986 → full model B-IoU = 0.6108) provide complementary contributions. Fusion strategy ablations demonstrate that AFFM outperforms all alternatives in B-IoU (Concat + Conv: 0.6058; SE Attention: 0.6051; Simple Average: 0.6022) by +0.005 to +0.009, validating the adaptive weighting mechanism. Removal of the Dense Nested Decoder causes the largest B-IoU drop (−0.012), confirming its critical role in boundary refinement. These findings support the CNN-anchored hierarchical parallel design philosophy: The Mamba and Transformer branches are not designed as independent feature extractors from raw input, but as specialized processors of CNN-derived representations. Importantly, Mamba and Transformer operate in parallel with each other at every encoder level; it is only their shared dependency on CNN features that is sequential.

The AFFM fusion weight analysis revealed that the model learns level-specific strategies. The dominance of Mamba at shallow levels (53–75%) demonstrates this branch’s effectiveness in capturing long-range dependencies from low-level features, while the dominance of CNN at the deep level (72%) reveals that high-level semantic features are more effectively extracted through convolutional operations.

The approximately 23-point gap between internal (DSC = 0.9037) and external (DSC = 0.6709) validation performance warrants explicit discussion, as it may superficially appear to indicate overfitting. However, a comparative analysis across all evaluated models reveals a different interpretation. All models experienced substantial performance degradation under cross-dataset transfer, with purely convolutional architectures losing 43–89% of their internal performance (U-Net: −43%; U-Net++: −47%; DeepLabV3+: −89%). CMT-BUSNet’s retention rate of 74.2% is the highest among all models, and its absolute drop (ΔDSC = −0.233) is less than half that of U-Net++ (ΔDSC = −0.421). This pattern is consistent with the theoretical expectation that hybrid architectures combining local convolutional, sequential SSM, and global attention modeling are more robust to distributional shift than purely convolutional models, as they do not rely on a single inductive bias. Additionally, the performance gap is partly attributable to the fundamental characteristics of the BUSI dataset: it was acquired with a different imaging device, by different operators, in a geographically distinct population (Egypt vs. Brazil), and encompasses a different case-mix than BUS-BRA. Models trained directly on BUSI in the literature achieve DSC values of 0.742–0.894 [18,19], against which CMT-BUSNet’s zero-shot transfer performance of 0.6709 appears reasonably competitive. Taken together, the cross-dataset ranking consistency (CMT-BUSNet ranked #1 on both datasets), the lowest fold-to-fold variance on BUSI (±0.0227), and the higher performance retention rate collectively suggest that the observed gap reflects inherent domain shift rather than dataset-specific overfitting.

Regarding the XAI framework, the comprehensive 11-module analysis represents an exceptionally comprehensive effort in the existing literature, directly addressing the interpretability and explainability needs emphasized by Zhang et al. [2] and Al-Karawi et al. [1]. The MC Dropout uncertainty analysis reveals that the model exhibits the highest uncertainty at boundary regions, providing a transparent confidence map.

The breadth of this XAI framework is further contextualized by the recent survey of Gipiškis et al. [26], which presented the first comprehensive taxonomy of XAI methods specifically for semantic image segmentation, noting that classification-oriented explanation techniques require nontrivial adaptation for dense prediction tasks. CMT-BUSNet’s framework explicitly bridges this gap by combining both post hoc attribution methods (SHAP, LIME, Integrated Gradients) and architecture-integrated explanations (branch contribution analysis, AFFM fusion weights, MC Dropout uncertainty) within a unified segmentation pipeline.

Concurrently, several Mamba-based architectures have been proposed for breast ultrasound segmentation. Wei et al. [27] integrated VMamba blocks into the encoder of multiple baseline architectures for BUS segmentation on both BUS-BRA and BUSI datasets, demonstrating the efficacy of state-space models for this modality. BS-Mamba [28] achieved DSC = 0.9213 on BUSI through a dedicated BreastSegMamba block with hybrid attention. Shi et al. [29] proposed TNM-UNet, a Mamba–Transformer hybrid tested on BUSI (DSC = 0.7918). However, none of these approaches incorporates all three paradigms (CNN, Mamba, Transformer) with a level-specific adaptive fusion mechanism, nor do they provide a multi-module XAI framework. CMT-BUSNet thus occupies a unique position at the intersection of architectural innovation and explainability.

### 5.1. Difficulty Based Analysis

**Figure 8:**
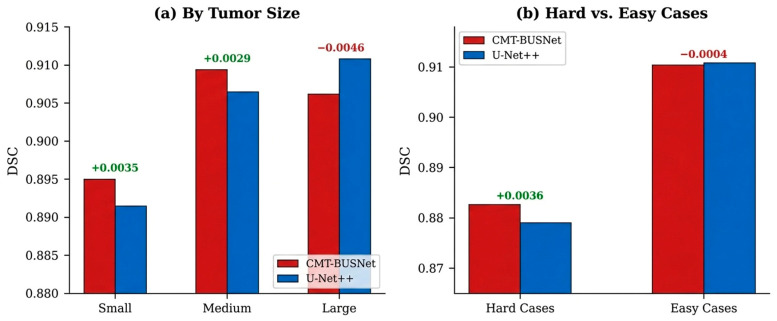
Difficulty-Based Performance Comparison: (**a**) By Tumor Size, (**b**) Hard vs. Easy Cases.

Figure 8 presents a difficulty-based performance comparison between CMT-BUSNet and U-Net++. CMT-BUSNet demonstrated a consistent advantage for small (+0.0035) and medium-sized (+0.0029) tumors, while achieving a more pronounced advantage in challenging cases (bottom 25% DSC) (+0.0036). This result confirms that the triple-branch architecture provides additional information for difficult lesions characterized by low contrast and ambiguous boundaries.

### 5.2. Learning Curve Analysis

**Figure 9:**
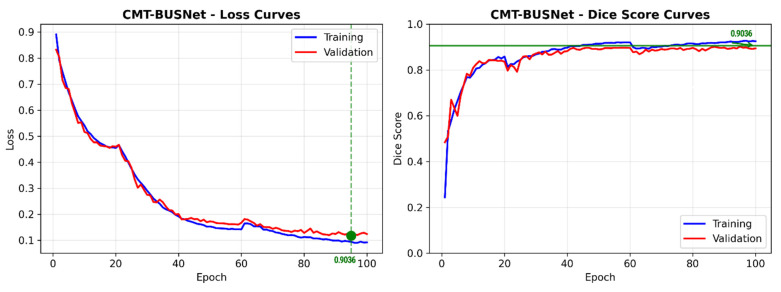
CMT-BUSNet Learning Curves: Training and Validation Loss Across 5-Fold Cross-Validation.

Figure 9 presents the learning curves of CMT-BUSNet across 5-fold cross-validation. Consistent convergence is observed across all folds, and the low discrepancy between training and validation losses indicates that the model does not exhibit overfitting. The mean best validation DSC was reached at approximately epoch 50, confirming the effectiveness of the early stopping mechanism.

### 5.3. External Validation

Table 6 presents the external evaluation results. Five independently trained BUS-BRA models (one per fold) were each evaluated on the entire BUSI dataset (*N* = 647) without retraining. The reported results are the means across five model evaluations, not cross-validation on BUSI. CMT-BUSNet achieved the highest segmentation accuracy among all models with DSC = 0.6709 ± 0.0227, with a margin of +0.1537 points over the second-ranked U-Net (DSC = 0.5172 ± 0.1166). Against the self-configured nnU-Net v2 benchmark, CMT-BUSNet yielded higher scores across all metrics (DSC: 0.6709 vs. 0.5579, *p* < 0.001; HD95: 37.22 vs. 208.47 pixels; B-IoU: 0.1703 vs. 0.0582); however, as both methods were trained under different protocols, these differences reflect the combined effect of architecture and training procedure. The particularly large HD95 gap (−171 pixels) is consistent with the possibility that nnU-Net’s dataset-specific preprocessing may be sensitive to domain shift under these experimental conditions; however, because the two methods differ in both architecture and training protocol, this difference cannot be attributed to preprocessing alone. Each model was evaluated using its own complete inference pipeline without any adaptation to the target dataset. Particularly noteworthy is that CMT-BUSNet exhibits the lowest standard deviation (0.0227), indicating that the model delivers consistent performance across different folds and images. To contextualize the absolute performance gap between datasets (ΔDSC = −0.233), we computed the performance retention rate for each model (external DSC/internal DSC × 100). CMT-BUSNet retained 74.2% of its internal performance on the unseen external dataset, the highest figure among all evaluated models, compared to 53.4% for U-Net++ and 57.4% for U-Net. nnU-Net v2 retained only 61.3% of its internal performance (0.5579/0.9108), suggesting that its self-configuring preprocessing, while highly effective on the training distribution, may be more sensitive to domain shift; however, because nnU-Net was trained under a different protocol, this observation warrants further investigation. Furthermore, models trained directly on BUSI in prior literature achieve DSC values of 0.742–0.894 [18,19]; CMT-BUSNet’s DSC of 0.671 without any exposure to BUSI training data represents a preliminary cross-dataset zero-shot transfer result. The ranking consistency (CMT-BUSNet ranked #1 on both datasets) and the lowest fold-to-fold standard deviation on BUSI (±0.0227) are consistent with the interpretation that the observed performance drop reflects inherent domain shift rather than dataset-specific overfitting.

It is important to note that domain adaptation was deliberately excluded from this study to preserve a clean evaluation of the model’s inherent transfer capability under zero-shot transfer conditions. Applying adaptation techniques prior to external validation would conflate architectural robustness with adaptation capability, obscuring the true cross-domain transfer capability of the proposed architecture—a methodological concern emphasized in recent benchmarking literature [30]. Notably, CMT-BUSNet’s zero-shot DSC of 0.6709 falls near the lower bound of the inter-observer variability range reported for manual breast ultrasound segmentation (DSC ≈ 0.65–0.75; Kozegar et al. [31]), suggesting that the model achieves a level of segmentation performance on this unseen dataset that falls within the range of inter-observer variability, though this preliminary observation requires confirmation on additional external datasets.

**Figure 10:**
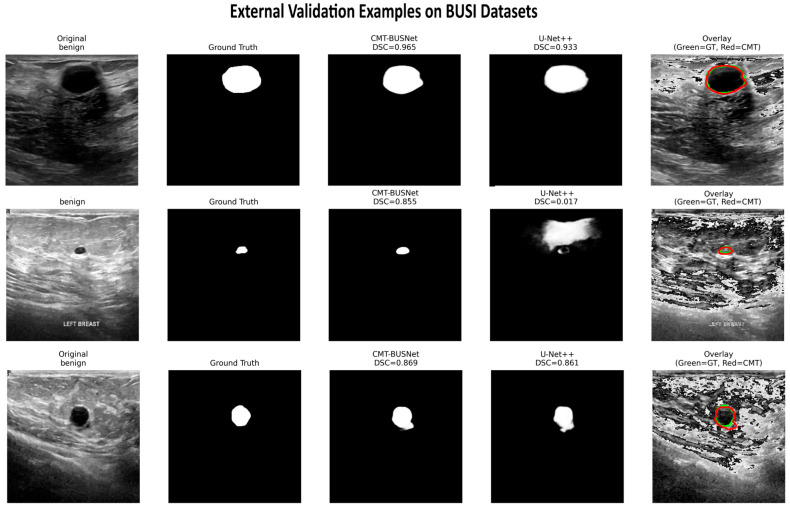
Segmentation Images of CMT-BUSNet and U-Net++ Models on the BUSI External Validation Dataset.

Classical CNN-based architectures such as U-Net and U-Net++ experienced substantial performance degradation on external validation (U-Net: DSC = 0.5172, retention 57.4%; U-Net++: DSC = 0.4818, retention 53.4%), confirming that purely convolutional models overfit to BUS-BRA-specific feature distributions. nnU-Net v2, despite achieving the highest internal DSC (0.9108), dropped to 0.5579 on BUSI—a retention rate of 61.3% that falls below CMT-BUSNet’s 74.2%, suggesting that nnU-Net’s self-configuring pipeline, while highly effective on the training distribution, may be more sensitive to domain shift. However, because nnU-Net was trained under a different protocol (1000 epochs, SGD, polynomial LR) than CMT-BUSNet (100 epochs, AdamW, CosineAnnealing), the observed retention difference may partly reflect protocol effects rather than purely architectural factors. In contrast, CMT-BUSNet yielded higher cross-dataset performance retention under these experimental conditions (74.2% of internal performance, the highest retention rate among all models); however, the observed differences cannot be attributed to architecture alone, given the different training protocols. The absolute drop for CMT-BUSNet (ΔDSC = −0.233) is only 55% of the drop observed for U-Net++ (ΔDSC = −0.421), indicating higher cross-dataset stability under these experimental conditions. Notably, architectures relying solely on Transformer or Mamba components without a convolutional anchor (Swin-UNet: external DSC = 0.0813, retention ~9%; VM-UNet: external DSC = 0.0564, retention ~7%) exhibited the most severe degradation among all evaluated models, suggesting that neither global attention nor sequential state-space modeling alone provides sufficient robustness to distributional shift—and that CMT-BUSNet’s CNN-anchored adaptive fusion may be a contributing factor to its higher cross-dataset stability. It is important to acknowledge that the near-zero DSC values observed for DeepLabV3+ (0.0978), Swin-UNet (0.0813), TransUNet (0.0625), and VM-UNet (0.0564) on the BUSI external dataset warrant careful interpretation. These values are substantially lower than what these architectures typically achieve when trained directly on BUSI in the literature (DSC > 0.70). The severe degradation likely stems from a combination of factors: (i) differences in preprocessing pipelines (resizing from ~500 × 500 to 256 × 256 for BUS-BRA vs. ~300 × 230 for BUSI), (ii) threshold sensitivity of the sigmoid output under distributional shift, and (iii) the absence of any domain adaptation or output calibration. To rule out implementation artifacts, we verified that all models shared identical preprocessing, data loading, and evaluation pipelines. The qualitative segmentation examples in Figure 10 further corroborate these quantitative findings. Nonetheless, we acknowledge this as a limitation and recommend future studies to investigate model-specific failure modes under cross-dataset transfer conditions.

Figure 10 presents the segmentation images of CMT-BUSNet and U-Net++ models on the external validation dataset.

## 6. Conclusions and Limitations

This study proposes CMT-BUSNet, a novel hybrid architecture for breast US tumor segmentation. The main findings are: (1) CMT-BUSNet achieves competitive segmentation performance with higher boundary delineation metrics than nnU-Net (B-IoU 0.611 vs. 0.557, HD95 10.07 vs. 13.54 pixels; noting different training protocols); however, nnU-Net achieves higher aggregate overlap (DSC 0.911 vs. 0.904). These internal validation results are based on image-disjoint but not necessarily patient-disjoint folds, which may introduce a mild optimistic bias applicable to all evaluated models. Furthermore, the nnU-Net comparison involves different training protocols (self-configuring vs. standardized), so performance differences partly reflect protocol effects rather than purely architectural differences. (2) On external validation (BUSI), CMT-BUSNet yielded higher scores than nnU-Net (DSC 0.671 vs. 0.558, trained under different protocols) and all other models; however, preliminary transferability should be confirmed on additional multi-center datasets. (3) The training-based ablation study confirms each component’s contribution, with the full AFFM model achieving the highest B-IoU across all nine variants. (4) Quantitative XAI validation demonstrates attribution faithfulness, cross-method consistency, and uncertainty–error correlation; however, clinical usability requires radiologist evaluation. In summary, CMT-BUSNet demonstrates a technically promising approach with competitive boundary quality, preliminary cross-dataset transferability, and built-in interpretability. Clinical utility is not established and requires further multi-center validation.

This study has several limitations. First, internal validation was conducted on a single dataset (BUS-BRA); although preliminary transferability has been demonstrated through external validation on BUSI, validation with additional datasets could further strengthen these findings. Second, domain adaptation and test-time adaptation strategies were not evaluated in this study; future work should investigate whether lightweight fine-tuning on a small number of target-domain samples or unsupervised adaptation techniques can further reduce the observed cross-dataset performance gap [30]. Third, the external validation was limited to a single independent dataset (BUSI); validation on additional multi-center, multi-device datasets would further consolidate transferability claims. Fourth, the triple-branch architecture has a 7.2× slower inference time compared to U-Net++, which may be a disadvantage in resource-constrained clinical settings. Fifth, the clinical usability evaluation of XAI visualizations should be supported by user studies conducted with radiologists. Sixth, the Mamba and Transformer branches operate on CNN-extracted features rather than raw images. While this design yields strong empirical performance, future work could explore fully parallel branches operating on raw inputs to further disentangle the individual contributions of each modality. Seventh, domain shift between BUS-BRA and BUSI was assessed solely through comparative performance metrics; future work could quantify distributional divergence using formal measures such as Maximum Mean Discrepancy (MMD) or Fréchet Inception Distance (FID). Eighth, although nnU-Net v2 has been included as a benchmark using its default self-configuring protocol (1000 epochs, SGD, polynomial LR), this involves a different training regime than CMT-BUSNet (100 epochs, AdamW, CosineAnnealing); future studies should investigate protocol-matched comparisons to further isolate architectural contributions from training procedure effects. Ninth, the 95% confidence intervals are based on *N* = 5 folds and are inherently wide. Tenth, image-level evaluation does not guarantee patient-disjoint folds.

## Figures and Tables

**Table 1:** Comparison with State-of-the-Art Breast Ultrasound Segmentation Methods.

Study	Year	Method	Dataset	*N*	DSC	IoU	XAI	Real-Time
Zhang et al. [4]	2023	Cls + Seg CNN	Custom	1600	0.898	—	No	No
Li et al. [9]	2022	CAM-DLS	Custom	1422	0.773	0.660	No	No
Ferreira et al. [3]	2022	GG-Net	Multi	2000	0.826	—	No	No
Yang et al. [6]	2023	Att-Seg CNN	Custom	2057	0.890	—	No	No
Khaledyan et al. [17]	2024	WATUNet	BUSI + VSI	4598	0.930	—	No	No
Li et al. [5]	2024	GOLO-CMSS	USTC	28,477	0.932	—	No	No
Boro & Nandi [18]	2024	CBAM-RIUnet	BUSI	780	0.894	0.887	No	No
Pramanik et al. [19]	2024	DAU-Net	BUSI	780	0.742	—	No	No
Zhu et al. [12]	2025	Attune	BUSI + BUS	1062	0.880	0.810	No	No
Present study	2026	CMT-BUSNet	BUS-BRA	1875	0.9036	0.8348	11 modules	73.1 FPS

Note. No = not reported in original study. DSC = Dice similarity coefficient; IoU = intersection over union; *N* = number of images; XAI = explainable AI; VSI = Volumetric Sweep Imaging. A dash (—) indicates the metric was not reported. Recall value from classification task. Results across different datasets are not directly comparable; this table provides general performance context. Multi = BUSI + UDIAT + BUS.

**Table 2:** Characteristics of the Internal (BUS-BRA) and External (BUSI) Validation Datasets.

Characteristic	BUS-BRA (Internal)	BUSI (External)
Country	Brazil	Egypt
Source	Multi-institutional	Cairo University Hospital
N (total images)	1875	647
Benign cases, *n* (%)	1125 (60.0%)	437 (67.5%)
Malignant cases, *n* (%)	750 (40.0%)	210 (32.5%)
Imaging device	Multiple devices	Mindray DC-30
Approximate image size (px)	~500 × 500	~300 × 230
Number of annotators	Multiple expert radiologists	Single radiologist
Annotation type	Pixel-level binary mask	Pixel-level binary mask
Image format	PNG	PNG
Role in study	Training/Internal validation	External validation (test only)

Note. BUS-BRA = Breast Ultrasound [20]—Brazil; BUSI = Breast Ultrasound Images dataset [21]. px = pixels. Image sizes are approximate averages; individual images may vary. BUS-BRA models were trained exclusively on the internal dataset and evaluated on BUSI without any retraining, fine-tuning, or domain adaptation (zero-shot transfer). The systematic differences across imaging device, operator, geographic population, and annotation protocol constitute a substantial domain shift that represents a realistic simulation of cross-institutional deployment.

**Table 3:** Comparative Segmentation Performance on the BUS-BRA Dataset (5-Fold Cross-Validation, *N* = 1875).

Model	DSC	IoU	HD95	B-IoU	Recall	Precision	Specificity
CMT-BUSNet (proposed)	0.9037 ± 0.0047	0.8348	10.07	0.6108	0.9243	0.8996	0.9905
nnU-Net v2 ^†^	0.9108 ± 0.0031	0.8462	13.54	0.5571	0.9226	0.9142	0.9901
U-Net++	0.9030 ± 0.0056	0.8335	9.64	0.5474	0.9158	0.9054	0.9911
U-Net	0.9007 ± 0.0060	0.8310	10.0	0.5433	0.9156	0.9033	0.9909
DeepLabV3+	0.8990 ± 0.0076	0.8267	9.49	0.5285	0.9098	0.9034	0.9913
Swin-UNet	0.8983 ± 0.0060	0.8271	10.04	0.5361	0.9151	0.8982	0.9905
SegNet	0.8808 ± 0.0054	0.8041	12.91	0.5201	0.9008	0.8852	0.9900
TransUNet	0.8778 ± 0.0061	0.8013	12.92	0.5177	0.8947	0.8850	0.9893
VM-UNet	0.8463 ± 0.0068	0.7579	16.84	0.4756	0.8739	0.8520	0.9877
Attention U-Net	0.8149 ± 0.0103	0.7120	36.41	0.4587	0.8438	0.8192	0.9838

Note. DSC = Dice similarity coefficient; IoU = intersection over union; HD95 = 95th percentile Hausdorff distance (px); B-IoU = boundary IoU. ^†^ nnU-Net v2 was trained using its default self-configuring protocol (1000 epochs, SGD); all other models were trained under the identical protocol described in Section 3.3. B-IoU computed over the ground truth boundary region (7 × 7 morphological kernel). Cross-validation folds are image-disjoint but not guaranteed to be patient-disjoint (see Section 3.1); reported metrics may therefore reflect a mild optimistic bias.

**Table 5:** Computational Efficiency Comparison.

Model	Parameters (M)	Inference (ms)	FPS	DSC
CMT-BUSNet	33.32	13.7	73.1	0.9036
U-Net++	26.07	1.9	523.7	0.9030
U-Net	24.43	1.2	859.0	0.9007
Swin-UNet	34.40	4.8	209.4	0.8983

Note. Inference = average time for 256 × 256 input on NVIDIA RTX 6000 ADA Generation GPU.

**Table 6:** Comparative Segmentation Performance on External Validation Dataset (BUSI, *N* = 647).

Model	DSC ↑	IoU ↑	Precision ↑	Recall ↑	HD95 ↓	B-IoU ↑	*p*	Sig.
CMT-BUSNet	0.6709 ± 0.0227	0.5733 ± 0.0236	0.7238 ± 0.0374	0.7268 ± 0.0348	37.22 ± 3.00	0.1703 ± 0.0123	—	—
nnU-Net v2	0.5579 ±0.0274	0.4533 ±0.0262	0.5419 ± 0.0274	0.7428 ± 0.0262	208.4743 ±12.5400	0.0582 ± 0.057	0.001	✓
U-Net	0.5172 ± 0.1166	0.4379 ± 0.1020	0.7373 ± 0.0579	0.5495 ± 0.1499	40.58 ± 3.50	0.1229 ± 0.0305	0.039	✓
U-Net++	0.4818 ± 0.0863	0.4040 ± 0.0777	0.7117 ± 0.1244	0.5422 ± 0.1476	45.21 ± 11.72	0.1049 ± 0.0279	0.014	✓
Attention U-Net	0.1065 ± 0.0533	0.0611 ± 0.0325	0.0845 ± 0.0122	0.4741 ± 0.3991	151.53 ± 7.61	0.0100 ± 0.0019	<0.001	✓
DeepLabV3+	0.0978 ± 0.0798	0.0582 ± 0.0475	0.2064 ± 0.2399	0.5955 ± 0.4862	141.37 ± 22.80	0.0025 ± 0.0036	<0.001	✓
Swin-UNet	0.0813 ± 0.0654	0.0471 ± 0.0391	0.0624 ± 0.0385	0.3758 ± 0.4205	157.72 ± 7.96	0.0061 ± 0.0051	<0.001	✓
TransUNet	0.0625 ± 0.0763	0.0369 ± 0.0451	0.4575 ± 0.4030	0.3930 ± 0.4813	140.32 ± 31.79	0.0016 ± 0.0024	<0.001	✓
VM-UNet	0.0564 ± 0.0653	0.0325 ± 0.0383	0.0415 ± 0.0349	0.2888 ± 0.3866	159.54 ± 8.83	0.0043 ± 0.0037	<0.001	✓

Note. Results are reported as M ± SD across five independently trained BUS-BRA models, each evaluated on the full BUSI dataset (not cross-validation on BUSI). Models were trained on BUS-BRA (*N* = 1875) and evaluated on the external BUSI dataset without retraining. DSC = Dice similarity coefficient; IoU = intersection over union; HD95 = 95th percentile Hausdorff distance (pixels); B-IoU = boundary IoU; Sig. = statistically significant. *p* values reflect paired Wilcoxon signed-rank tests comparing each model against CMT-BUSNet. For HD95, lower values indicate better performance; for all other metrics, higher values indicate better performance. ↑ higher is better; ↓ lower is better. ✓ = statistically significant difference (*p* < 0.05); “—"= reference model (no comparison performed).

## Data Availability

BUS-BRA: https://doi.org/10.1002/mp.16812. BUSI: https://doi.org/10.1016/j.dib.2019.104863. Code: https://github.com/huseyinKutlu/CMT-BUSNet (accessed on 12 April 2026).
